# Environmental persistence of nontyphoidal *Salmonella* in an urban informal settlement in Nairobi, Kenya

**DOI:** 10.1371/journal.pone.0321760

**Published:** 2025-04-28

**Authors:** Collins K. Kebenei, David Onyango, Kelvin Kering, Cecilia Mbae, Susan Kavai, Michael Muraya, Celestine Wairimu, Georgina Odityo, Kristin Weber, Michael Pietsch, Tanja Pilz, Oliver Drechsel, Andrea Thürmer, Torsten Semmler, Stephan Fuchs, Sandra Simon, Antje Flieger, Lothar H. Wieler, Samuel Kariuki

**Affiliations:** 1 Centre for Microbiology Research, Kenya Medical Research Institute, Nairobi, Kenya; 2 School of Physical and Biological Sciences, Maseno University, Maseno, Kenya; 3 Department of Infectious Diseases, Robert Koch Institute, Wernigerode, Germany; 4 Genome Competence Center, Robert Koch Institute, Berlin, Germany; 5 Digital Global Public Health, Hasso Plattner Institute, University Potsdam, Potsdam, Germany; 6 Drugs for Neglected Diseases Initiative, Nairobi, Kenya; University of Illinois Urbana-Champaign College of Veterinary Medicine, UNITED STATES OF AMERICA

## Abstract

Non-typhoidal Salmonella (NTS) presents a considerable health threat to children in low-resource settings, where clean water, sanitation, and hygiene are often inadequate. However, the environmental factors influencing NTS persistence and spread remain poorly understood. We utilized a case-control approach to investigate environmental factors associated with NTS infection in children living in Nairobi’s informal settlements between August 2022 and July 2023. Stool samples were collected from febrile children, with or without diarrhea, who visited healthcare facilities. The study included 42 laboratory-confirmed NTS-positive cases and 42 NTS-negative children from the same community. Environmental samples, including drinking water, open drains, soil, and household effluent, were collected from both case and control households, in addition to raw sewage from main sewer-line convergence points. Conventional microbiological culture and quantitative Polymerase Chain Reaction techniques were employed for NTS detection, with genomic sequencing used for strain characterization. Environmental samples from case households showed a higher NTS contamination rate of 33.3% (42/126) compared to control households of 7.2% (9/126). Higher odds of NTS infection in children were associated with household environmental factors, particularly exposure to household effluent (OR = 7.7, 95% CI: 2.18–34.82, *p* = 0.0005), drinking water (OR = 6.4, 95% CI: 1.57–37.76, *p* = 0.0055), and soil (OR = 5.4, 95% CI: 1.01–54.28, *p* = 0.0485). Genomic analysis revealed a common strain, Salmonella Enteritidis ST11, in clinical and environmental isolates. These findings highlight the plausible role of the household environment as a reservoir for NTS, perpetuating infection cycles within the community. Addressing this challenge requires a multifaceted approach, including improved sanitation infrastructure, environmental monitoring, and integrated public health interventions to reduce NTS exposure and transmission in high-risk populations.

## Introduction

Non-typhoidal *Salmonella* (NTS) infection is associated with self-limiting gastroenteritis in many developed countries. However, in Sub-Saharan Africa, it is linked to severe life-threatening bloodstream infections, especially in children under five years of age [1]. The disease is linked to over 3.4 million cases in Sub-Saharan Africa, with an annual mortality of about 23% [2,3]. Often, those with immunosuppression, malnourishment, and concurrent malaria infections are among the people at risk. Conversely, children are the most affected, accounting for over 63% of NTS cases in Sub-Saharan Africa [2,4]. The high risk of infection is particularly pronounced for children because of their underdeveloped immune and weak gastrointestinal systems [4], coupled with their indiscriminate outdoor activities, potentially exposing them to contaminated environments [5].

Although several strains of *Salmonella enterica* are associated with NTS infection, in sub-Saharan Africa, *Salmonella typhimurium* (*S. typhimurium*) and *Salmonella enteritidis* (*S. enteritidis*) are the main serovars known to cause NTS disease [1,2]. The widespread and persistence of NTS strains in sub-Saharan Africa, particularly in the communities living in resource-limited settings, is influenced by various factors, including poor living conditions, inadequate sanitation, limited access to clean water, improper sewage disposal, poor food handling practices, and consumption of contaminated foods [3,6,7]. These challenges are further compounded by resource scarcity and inadequate healthcare infrastructure, leading to delayed diagnosis and treatment of severe infections. In addition, storm-triggered floods may play a pivotal role in spreading fecal waste to different ecological compartments, thereby disseminating NTS pathogens in the community [5,8]. Given these multiple layers of risk, understanding how environmental contamination perpetuates NTS persistence and transmission is vital to controlling its spread.

This study aimed to investigate NTS’s distribution and seasonal variation, focusing on its occurrence among children under five years and their immediate household environments in an informal settlement in Nairobi, Kenya. Understanding these dynamics is important to comprehend the role the environment plays in the persistence and dissemination of NTS in disease-endemic settings, ultimately enabling the development of effective strategies to manage and prevent NTS infections in such regions.

## Materials and methods

### Study site

Mukuru informal settlement covers an area of roughly 263 hectares and is home to an estimated 150,000 people. The settlement is located about 20 kilometers east of Nairobi City (latitude 1° 18′ 17′ S and longitude 36° 53′ 6′ E) [9]. It is characterized by overcrowding, inadequate sanitation practices, improper sewer disposal, and limited access to clean drinking water, thereby making it a hotspot for NTS infection [2,3]. During the rainy season, overcrowded housing units are prone to flooding from stormwater and open sewers, increasing the risk of disease transmission [6].

### Study design

This was a case-control study conducted between the 1st of August 2022 and the 31st of July 2023, involving the collection of stool samples from children aged 5 years and below experiencing diarrhea with or without fever. The study included 42 laboratory-confirmed NTS-positive cases and 42 NTS-negative controls. Controls were randomly selected from households located 100 meters from the case household. Children were recruited from participating healthcare facilities, including Medical Missionaries of Mary-Mukuru Health Centre, Reuben NMS Hospital, City Council Clinic, Njenga NMS Hospital, and Mama Lucy Hospital, all within the Mukuru informal settlements in Nairobi County. Written informed consent was obtained from parents or guardians before enrollment. Positive cases were followed up, and environmental samples, including drinking water, open drains/effluent, and soil, were collected to screen for NTS in different ecological niches and assess potential exposure sources. Similar environmental samples were collected from the control households for comparison. To further explore additional contamination pathways, 16 raw sewer samples (untreated human waste collected directly from the sewage system) were collected quarterly between August 2022 and July 2023. Secondary data on average monthly rainfall patterns were obtained from the Kenya Meteorological webpage to support the analysis [10].

### Sample size calculation

This study utilized a sample size of 42 confirmed NTS-positive cases, determined using the formula $n=\frac{[z^{2}\;p\;q]}{e2}$ [11] and based on a previously reported NTS isolation rate of 2.8% within the study population [3]. The isolation rate corresponds to an estimated attribute proportion (p) of 0.028. A confidence level of 95% (z = 1.96) and an alpha level (e²) of 0.05 were selected following Kothari’s guidelines[11]. To assess potential environmental reservoirs, three environmental samples (soil, drinking water, and open drain) were collected from each case household, resulting in a total of 126 environmental samples. An equal number of environmental samples were also collected from control households for comparative analysis.

### Stool sample collection

Fecal or rectal swabs were collected using sterile cotton-tipped swabs and were transported in a Cary-Blair transport media (Oxoid, Basingstoke, UK). The samples were shipped in a cold box to the Kenya Medical Research Institute’s (KEMRI) Center for Microbiology Research facility for processing.

### Environmental sample collection

To screen for the presence of NTS strains in different ecological settings, we collected samples of drinking water, household open drains/effluents, and soil from the households of NTS-positive children (index case) and randomly selected control households, following Raj’s protocol [12]. Raw sewer samples were also collected from the main site of convergence of the sewerage systems in the study area. In addition, we collected 100 g of soil from children’s play areas in residential neighborhoods, which were devoid of apparent fecal contaminants. The collected samples were placed in sterile Whirl-Pack bags and transported in an ice-packed cooler box to the laboratory at the Centre for Microbiology Research within Kenya Medical Research Institute (KEMRI) for further processing.

### Stool sample processing

As soon as the stool samples arrived at the laboratory, they were processed as described [2,6]. To summarize, fecal samples in the Cary-Blair media (Oxoid, Basingstoke, UK) were enriched in Selenite F broth (Oxoid, Basingstoke, UK) and incubated at 37°C for 24 hours. An aliquot of the enriched Selenite F broth was cultured on MacConkey (Oxoid, Basingstoke, UK) and Xylose Lysine Deoxycholate agar (Oxoid, Basingstoke, UK), and incubated for 24 hours at 37°C. *Salmonella* suspects were sub-cultured on Mueller-Hinton agar (Oxoid, Basingstoke, UK) and identified by biochemical test and Analytical Profile Index (API) 20E strips. Confirmation was performed using *Salmonella* strain-specific serological tests based on the Kaufman-White Scheme [13].

### Environmental samples processing

Environmental samples were processed as previously described [14] with minor adjustments. To summarize, 100 g of soil sample was pre-processed by mixing in 200 ml of buffered peptone solution for 30 minutes at 150 rpm. All the samples were then split into two portions. The first 25 ml of the environmental samples were enriched in Rappaport-Vassiliadis broth (Oxoid, Basingstoke, UK) and incubated at 41°C for 24 hours. An aliquot of the enrichment broth was streaked onto MacConkey (Oxoid, Basingstoke, UK) and Xylose Lysine Deoxycholate agar (Oxoid, Basingstoke, UK) and incubated for 24 hours at 37°C. Confirmation for suspected NTS strains was using *Salmonella* strain-specific serological tests based on the Kaufman-White Scheme [13].

Consequently, metagenomic DNA was extracted from all the remaining portion of environmental samples using the ZymoBiomics DNA Miniprep Kit (Zymo Research, USA), following the manufacturer’s instructions, with DNA quantified using a NanoDrop spectrophotometer (ND-2000, Thermo Fisher, Waltham, MA, USA), and prepared for downstream molecular analysis.

### Screening for NTS serovars from environmental samples by multiplex real-time qPCR

This study used the multiplex quantitative polymerase chain reaction (qPCR) technique to screen for *S.* Typhimurium and *S.* Enteritidis using published primers (Table 1) previously described by Maurischat (32). PCR amplification was performed in a magnetic induction cycler (Mic) qPCR from Bio molecules Systems. The PCR mix contained 12.5 µl of 2X Luna Universal Probe qPCR Master Mix M3004 (Biolabs, New England), 2 µl of both sets of forward and reverse primers (at a concentration of 0.5 µM) (Biolabs, New England), 3.5 µl of DNA-free molecular water, and 5 µl of DNA template to a final reaction volume of 25 µl. The following amplification conditions were used: initial denaturing at 95°C for 1 minute, followed by 40 cycles of 95°C for 15 seconds, 59.5°C for 30 seconds, and a final extension of 72°C for 15 seconds. All samples were analyzed in duplicate, and each experiment contained positive and negative controls to guarantee the precision and validity of the experimental findings, respectively. A cut-off threshold of Ct <36 was applied to distinguish a positive outcome.

**Table 1:** *Salmonella enteriditis* and *Salmonella typhimurium* multiplex qPCR primers for amplification and characterization of serovars.

Gene	Primer	Sequence	Annealing Temp (°C)	References
*safA*	*safA-F* *safA_R* *safA-P*	GGTTGCTAACACGACACTG TGGGGCATTGGTATCAAAG **FAM-**CTCCTCCCATTCCACATTTGCG**-BHQ1**	59.5	[15]
*fliA-IS200*	*fliA-F* *fliA-R* *fliA-P*	CATTACACCTTCAGCGGTAT CTGGTAAGAGAGCCTTATAGG **HEX-**CGGCATGATTATCCGTTTCTACAGAGG**-BHQ1**	59.5	

The table lists the gene, primer sequences, annealing temperature, and reference for the primers used. The safA and fliA-IS200 genes were utilized to detect *S.* Enteritidis and *S*. Typhimurium, respectively. It also provides information on the fluorescent dyes used in the qPCR assay (FAM: 6-carboxyfluorescein and HEX: hexachlorofluorescein; and their corresponding quenchers are BHQ1).

### Whole genome sequencing and analysis

At the end of the study, all non-typhoidal (NTS) strains isolated from clinical cases and their corresponding isolates from the environmental sources were subcultured and grown overnight in nutrient agar (Oxoid, Basingstoke, UK) for DNA extraction and whole genome sequencing. These included two samples identified as *S. enteritidis* from environmental sources (drinking water and open drain/effluent), and their corresponding clinical cases. Two NTS isolates (*S. enteritidis* and *S. typhimurium*) from environmental samples could not be revived and were excluded from further analysis. DNA was extracted using Quick-DNA Fungal/Bacterial Kits (Zymo Research) and shipped on ice to the Genomic Competent Centre, Robert Koch Institute in Germany for sequencing.

Sequencing libraries were prepared following the Nextera XT prep kit (Illumina, Inc, San Diego, CA, USA) and sequenced on an Illumina NextSeq 2000 system using a P3 cartridge with an output of 150 bp paired-end reads. FastQC v0.12.1 [16] was used to assess the quality of the raw reads, with trimming of low-quality bases done using Fastp v0.23.4 [17]. Kraken v2.1.3 [18] was used for preliminary taxonomic classification of the raw reads from environmental samples based on the standard kraken2 database_nt. Genomic mapping was performed using bowtie2 v2.5.4 [19], with SAMtools v1.20 [20] used to generate consensus sequences from the aligned reads. The reference genome LT2 (genome assembly ASM694v2) was used for genome mapping and variation calling. Further, Snippy v4.6.0 [21] was used to carry out multiple variant calling. Repetitive sequences were filtered out using snippy tools and Snps-sites v2.5.1 [22], while Gubbins v3.3.5 [23] was used to identify loci with elevated densities of base substitutions. SPAdes v4.0.0 was used for *de novo* genome assembly [24], while Prokka v1.14.5 was used for genome annotation [25]. A maximum likelihood (ML) phylogenetic tree was inferred from the core-genome SNP alignment using RaxML-ng v1.2.2 [26], with 1000 bootstraps iterations. The resulting tree was visualized with Figtree v1.4.4 [27].

*Salmonella* strain typing was performed using seqsero2v1.3.1 [28]. In addition, MLST v2.23.0 [29] was used to determine the sequence type based on the Achtman seven-gene MLST scheme, which includes the seven housekeeping genes *aroC, dnaN, hemD, hisD, purE, sucA, and thrA*.

ABRicate v1.0.1 [30] was utilized to screen for plasmid replicons, virulence factors, and antimicrobial resistance genes using the PlasmidFinder database [31], VFDB [32], and CARD database [33], respectively. Further, a custom-built Python script was developed to process ABRicate output files, utilizing the os, glob, pandas, subprocess, and re modules. This in-house script aggregated data on gene presence and coverage to generate summary tables for antimicrobial resistance genes, virulence factors, and plasmid-associated genes. Heatmaps with hierarchical clustering were subsequently created using the pheatmap (v1.0.12) [34], dplyr (v1.1.4) [34], RColorBrewe**r** (v1.1.3) [35], and Cairo (v1.6.2) [36] packages, providing visual representations of the aggregated summary tables.

NTS isolates from environmental and clinical sources were classified as identical types only when they shared the same sequence type and demonstrated genetic congruence across key genetic determinants, including antimicrobial resistance genes, virulence factors, and plasmid replicons.

### Data management and analysis

All the study data were recorded in Microsoft Excel and Epicollect software. Tables and graphs were used to present data on the distribution of NTS in different ecological niches as well as seasonal trends in their occurrence. The Pearson correlation test was used to evaluate the correlations in the occurrence of NTS in the case and control households. The chi-square test was used to determine the association between NTS detection in diverse ecological niches and the incidence of infection among the study subjects. All the statistical analysis was performed using R-statistics [37], with a significance level of p < 0.05.

### Ethical considerations

Nairobi County Health Services granted permission to conduct the study in Mukuru, with permit number NCCG/DHS/REC/25. Ethical approval was obtained from the Scientific and Ethics Review Unit (SERU) of Kenya Medical Research Institute, with reference number KEMRI/SERU/CMR/P00205-04-2022/4497. A research license was acquired from the National Commission for Science, Technology, and Innovation (NACOSTI), with reference number 485239.

## Results

### Detection of NTS among children under 5 years and their household environment

During the study period, we collected 2,675 samples from recruited cases, of which 1.57% (42/2,675) tested positive for NTS, with *Salmonella* Enteritidis and *Salmonella* Typhimurium showing almost similar detection rates at 52.4% (22/42) and 47.6% (20/42), respectively. We consequently followed the 42 NTS-positive cases and an equal number of control households, collecting 252 environmental samples from the case and control households. Tragically, one child among the 42 NTS-positive cases developed severe complications and succumbed to an NTS-related illness during the study.

Environmental samples from case households (n = 126) showed varied degrees of NTS detection, with effluent having the highest rate of NTS detection (33.3% for *S. enteritidis* and 11.9% for *S. typhimurium*), compared to drinking water (26.2% for *S. enteritidis* and 7.1% for *S. typhimurium*), and soil (14.3% for *S. enteritidis* and 21.4% for *S. typhimurium*) (Table 2). Notably, four NTS isolates (one *S. typhimurium* and three *S. enteritidis*) were recovered by culture from case household drinking water and open drains, respectively. In contrast, environmental samples from control households (n = 126) showed a low NTS detection rate (7.14%), with all positive samples identified as *S. enteritidis* in drinking water (7.1%), effluent (9.5%), and soil (4.8%) (Table 2).

**Table 2:** Detection of *Salmonella enteritidis* and *Salmonella typhimurium* in case and control household environmental samples.

Sample type	No. of Samples collected (case + control)	Case household			Control household			Odds ratio	CI lower	CI upper	p-value
		*Salmonella enteritidis*	*Salmonella typhimurium*	Total NTS positive	*Salmonella enteritidis*	*Salmonella typhimurium*	Total NTS positive				
Drinking Water	**84**	11 (26.2%)	3 (7.1%)	14 (33.3%)	3 (7.1%)	0	3 (7.1%)	6.5	1.57	37.76	0.0055
Effluent	**84**	14 (33.3%)	5 (11.9%)	19 (45.2%)	4 (9.5%)	0	4 (9.5%)	7.7	2.18	34.82	0.0005
Soil	**84**	6 (14.3%)	3 (7.1%)	9 (21.4%)	2 (4.8%)	0	2 (4.8%)	5.4	1.01	54.28	0.0485
**Total**	**252**	**31 (24.6%)**	**11 (8.7%)**	**42 (33.3%)**	**9 (7.1%)**	**0**	**9 (7.1%)**				

The odds ratio analysis indicated a significant association between household environment and NTS infection in children, identifying key potential sources of exposure. Compared to control households, NTS detection was significantly higher in drinking water, effluent, and soil. Specifically, the odds of NTS detection in drinking water were approximately six times higher (OR = 6.4, 95% CI: 1.57–37.76, p = 0.0055), with effluent and soil showing a similar elevated odds (OR = 7.7, 95% CI: 2.18–34.82, p = 0.0005) and (OR = 5.4, 95% CI: 1.01–54.28, p = 0.0485), respectively (Table 2).

A total of 16 samples from four sewer convergent points were collected quarterly for one year during the study period to investigate their potential for environmental contamination. NTS was detected by PCR in all the sewer samples analyzed, indicating the persistence of NTS in the sewage system. Notably, *S. enteritidis* was detected at a higher rate (75%) than *S. typhimurium* (68.8%), suggesting that the sewer system could be a possible source of environmental contamination.

### Phylogenetic relatedness of NTS isolates from the indexed case and the environmental samples

Among the four NTS isolates obtained from the environmental samples and preserved, only two (one from drinking water and one from an open drain) could be successfully revived through culture. These two revived isolates, originating from the same index case household environment, were identified as *S. enteritidis* ST11, matching the sequence type found in the clinical isolate from the case.

**Fig 1:**
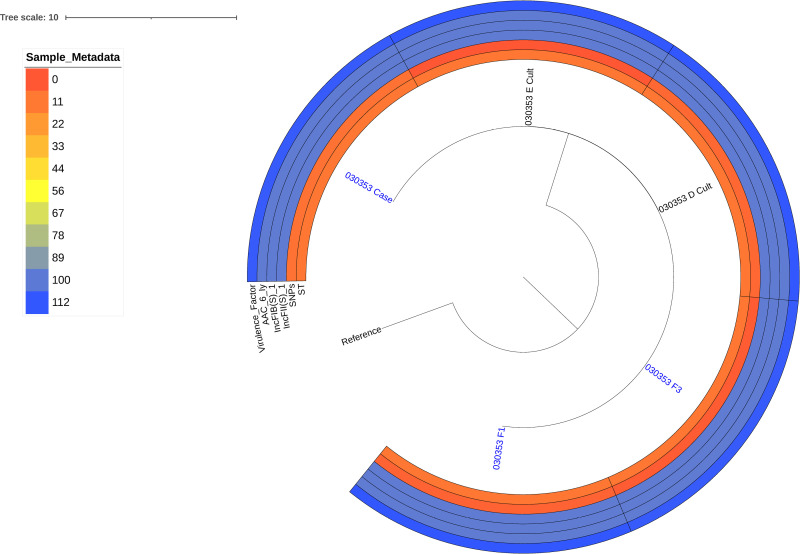
Phylogenetic tree of *S.* *enteritidis* isolate recovered from the index case and its immediate household environment. Tip labels represent sample and isolate identifiers. The numbers highlighted indicate the isolates obtained from the index case during the active disease period and the subsequent follow-ups. Isolates denoted as 030353_D_cult and 030353_E_Cult indicate the isolates recovered from the index case drinking water and surrounding open drains, respectively. The number denoted as 030353_Case indicates the first isolate obtained from the index case during the acute disease period, while those denoted as 030353_F1 and 030353_F3 indicate the isolates recovered from the index case during the first and the third follow-ups, respectively. The accompanying metadata heatmap provides additional genomic information on sequence type (ST), single nucleotide polymorphisms (SNPs) relative to the reference genome, plasmid replicons (*IncFII*(S)_1 and IncFIB(S)_1), and a total number of virulence factors in each isolate. The heatmap employs a color gradient with lighter shades indicating lower values and darker shades indicating higher specific attributes.

Notably, the isolates recovered from the clinical case (030353_Case, 030353_F1, and 030353_F3) and those from the environmental sources (030353_D_cult and 030353_E_cult) were closely related, with a maximum SNP difference of 9 between them (Fig 1 and S1 Table).

### Distribution of antimicrobial resistance genes, virulence factors, and plasmid replicons

A comprehensive analysis of sequenced NTS isolates revealed a consistent set of 27 antibiotic resistance genes (ARGs), with gene coverage ranging from 98% to 100% among isolates (S2 Fig). These ARGs predominantly encoded for efflux pumps (*acrA, acrB, acrD, baeR, cpxA, mdsA, mdsB, mdsC, mdtB, mdtC, mdtK, msbA, emrA, emrB, emrR,* and *tolC*) and regulatory proteins (*marA, baeR, kdpE, H-NS, CRP, sdiA*, and *golS*), illustrating a robust repertoire of resistance determinants shared across the environmental and clinical isolates of *S. enteritidis* strains from the same household. In addition to these general resistance mechanisms, specific ARGs that may confer resistance to diverse antimicrobial agents were identified. This includes *AAC (6’)-Iy* which inactivates aminoglycosides, *ampH*, which provides resistance to β-lactams, and *bacA*, which enhances resistance to bacitracin. Furthermore, the detection of *yoji,* mediating resistance to antimicrobial peptides, underscores the diversity of antimicrobial resistance strategies within these isolates.

In addition, plasmid replicons *IncFII(S)_1* and *IncFIB(S)_1*, both exhibit 100% genome coverage across all isolates. These replicons are particularly significant for their association with antimicrobial resistance and virulence determinants critical for bacterial survival and pathogenesis (S3 Fig).

The comparative analysis of virulence genes across *S. enteritidis* strains from environmental and clinical sources revealed a diverse yet consistently similar set of pathogenicity determinants, with gene coverage ranging from 88% to 100% across all isolates (S4 Fig). Notably, this study identified a wide array of virulence genes, including those encoded within *Salmonella* Pathogenicity Islands (SPIs), particularly SPI-1 and SPI-2, central to *Salmonella*’s virulence and pathogenesis.

Adhesion-related genes (*csgA-G*, *fimC-I*, *lpfA-E*, *misL*, *pefA-D*, and *sinH)*, crucial for fimbriae production and host cell attachment, were consistently identified across all strains. These adhesion genes are fundamental for initial colonization and persistence within the host environment reflects the pathogen’s ability to establish itself effectively. This was complemented by invasion-related genes (*invA-J, sipA-D, sptP, orgA,* and *prgH-K*), predominantly encoded within SPI-1, which facilitate the incursion of host cells and the early stages of infection through their role in the Type III Secretion System (T3SS-1). These invasion genes orchestrate cytoskeletal rearrangements and membrane ruffling, enabling bacterial uptake by epithelial cells.

Similarly, a wide collection of toxin-producing genes (*sopA-D, sopD2, sopE2*, and *slrP*) were identified, many of which are SPI-1-encoded effectors. These toxins play pivotal roles in tissue damage and modulation of host immune responses, aiding in the establishment and progression of infection. In parallel, genes linked to immune evasion, including *avrA, sodCI,* and *mig-14*, were consistently detected across all the isolates, emphasizing the pathogen’s ability to circumvent host defenses and enhance intracellular survival. Notably, the identification of SPI-2-associated genes (*ssaB-C, spiC, sseA-L, sifA-B,* and *pipB2*), which encode components of T3SS-2, underscores their role in enabling intracellular survival, replication, and systemic dissemination by maintaining the integrity of the Salmonella-containing vacuole (SCV).

We further revealed a pronounced metabolic versatility among the isolates from clinical and environmental samples, particularly through the detection of iron acquisition genes (*entA-B* and *fepG*) that are critical for survival in the iron-limited environments within the host.

The T3SS repertoire, encompassing structural genes associated with SPI-1 (*spaO-S, orgB-C, sicA,* and *sicP*) and SPI-2 (*ssaB, ssaC-V,* and sseA-L) as well as effector genes (*pipB-B2, sifA-B, sscA-B, steA-C, sseK1,* and *sspH2*) mostly encoded within SPI-2, except for the *steA_C* (SPI-1). This highlights the strains’ reliance on sophisticated molecular machinery to deliver effector proteins into host cells and modulate host cellular processes to establish infection and ensure intracellular survival.

Additionally, the detection of *Salmonella* plasmid-associated virulence genes (*spvB*, *spvC*, and *spvR*) across all isolates underscores the strains’ potential for systemic infection, driven by increased toxin production and enhanced invasiveness. The presence of stress response genes (*mgtB-C* and *ratB*) and outer membrane protein genes (*ompA* and *rck*) across all isolates further highlights their ability to maintain structural integrity, withstand hostile environmental conditions, and resist host immune responses (S2 Table).

Together, these findings demonstrate that *S. enteritidis* strains from environmental and clinical samples possess a versatile and comprehensive pathogenicity profile. The integration of SPI-1 and SPI-2-encoded genes alongside other virulence determinants equips these strains to adapt to diverse environments and persistently sustain infection, thus emphasizing their significant public health threat.

### Seasonal variation of environmental non-typhoidal *Salmonella*

**Fig 2:**
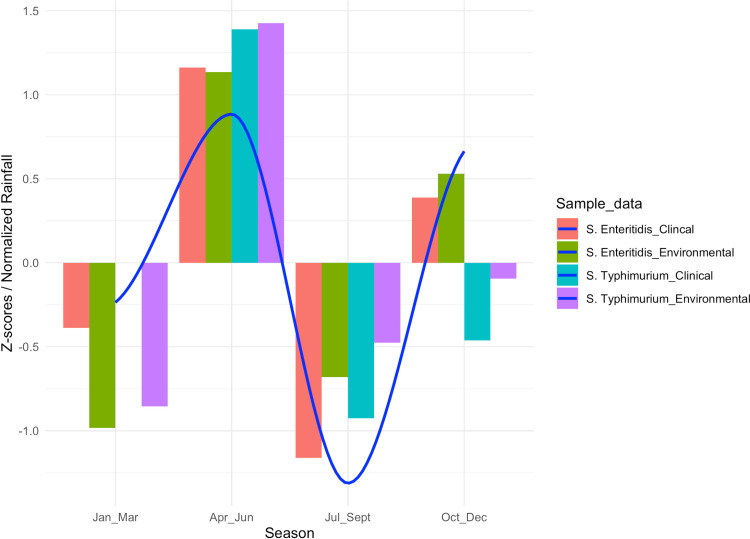
This graph illustrates the relationship between the detection of NTS strains (*S.* *typhimurium* and *S. enteritidis*) in both environmental and clinical samples (index children’s cases) throughout the year in Mukuru informal settlements, Nairobi, Kenya. The clinical strains are represented as *S.* Enteritidis_Clinical and *S.* Typhimurium_Clinical, while the environmental strains are labeled as *S.* Enteritidis_Environmental and *S.* Typhimurium_Environmental. The x-axis denotes the seasons, and the y-axis represents the Z-score, which measures deviations from the overall average in standard deviations. A significance threshold of Z = 1.96 is included. All Z-scores fall below this threshold, indicating no statistically significant seasonal variation. Additionally, the blue curve overlays the graph to represent the seasonal rainfall pattern, included as a reference to assess its potential association with the detection trends of NTS strains. Abbreviations: Jan = January, Mar = March, Apr = April, Jun = June, Jul = July, Sep = September, Oct = October, Dec = Decembermbe.

The detection of NTS (*S. enteritidis* and *S. typhimurium*) from the environment showed a marked increase during the rainy months of April to June and October to December, compared to the relatively dry-to-low rainfall periods of January to April and July to September. Comparably, NTS incidences in the community mirrored this seasonal variation, with a higher disease burden observed during the wetter months (Fig 2). However, further statistical interrogation indicates no significant variation in the detection of NTS across the seasons, as all Z-scores fall below the significance threshold of 1.96 at a 95% confidence interval. Specifically, for *S. enteritidis* and *S. typhimurium* from clinical samples, the Z-score was −0.39 and 0 in the first quarter (January–March), followed by 1.16 and 1.39 in the second quarter (April–June), −1.16 and −0.93 in the third quarter (July–September), and 0.39 and −0.46 in the last quarter (October–December) respectively, while *S. enteritidis* and *S. typhimurium* from the environmental samples recording a Z-score of −0.98 and −0.86 in the first quarter of the year, followed by 1.13, and 1.43 in the second quarter, −0.68, and −0.48 in the third quarter, and finally 0.53 and −0.10 in the last quarter, respectively over the same period (Fig 2).

## Discussion

The persistence and dissemination of NTS in the environment present a considerable public health challenge, especially in resource-constrained informal settlements where the risks of disease transmission are notably high. This study investigated the impact of environmental contamination on detecting and isolating NTS pathogens from children under five years and their immediate household environments in an endemic informal settlement in Kenya.

The study observed consistently high detection rates of NTS all year round, with notable peaks from both environmental and clinical sources occurring in April–June and October–December (Fig 2), coinciding with periods of relatively high rainfall. It should be noted that while the detection of NTS DNA from the environment is likely indicative of the potential presence of the corresponding NTS strain type. This approach, while efficient, may overestimate the actual prevalence of viable NTS pathogens in the environment due to the inherent inability of DNA-based techniques to discriminate between DNA originating from viable bacterial cells, non-viable bacteria, or extracellular DNA persisting in the environment after cell death [38]. However, the detection of NTS strains, as rendered in this study, presents a quick and efficient way to screen for the possible presence of putative NTS strains in the environment.

In this study, we identified a significant association between the frequency of NTS infections in children and its detection in various environmental samples, highlighting the environment as a potential source of NTS exposure to children. Specifically, the presence of NTS was notably higher in drinking water (OR = 6.4, 95% CI: 1.57–37.76, p = 0.0055, effluent (OR = 7.7, 95% CI: 2.18–34.82, p = 0.0005), and soil (OR = 5.4, 95% CI: 1.01–54.28, p = 0.0485) samples collected from case households compared to control households. These findings suggest that household environmental contamination may play a crucial role in sustaining NTS transmission, posing an elevated risk to vulnerable populations within the community. Children may be particularly vulnerable to NTS infections, given their underdeveloped immune systems [4] and frequent exposure to contaminated environments during outdoor activities [5] Our results build upon previous studies from Mukuru’s informal settlements, which have documented a high burden of NTS among children [2,3,6,7,39]. By demonstrating the potential contribution of environmental reservoirs to NTS persistence and spread, our study highlights a critical public health concern. The presence of NTS in household drinking water and effluent points to widespread contamination of water systems, likely contributing to the continuous circulation of NTS within this community. These results are consistent with broader research linking environmental contamination to infectious disease transmission in resource-limited settings [5–7,40], emphasizing the urgent need for targeted interventions to mitigate environmental exposure risks.

The substantially higher detection rates of NTS in environmental samples from case households compared to controls indicate a silent connection between household contamination and infection in children, contributing to the persistent burden of NTS infections throughout the year. However, our findings do not establish the directionality of disease transmission, highlighting the need for future research to trace NTS sources and model its transmission pathways to better understand its spread within the community.

Although the sources and transmission pathways of NTS in sub-Saharan Africa remain poorly characterized [41,42], our findings reveal a compelling genetic connection between isolates from index cases and their household environments. These isolates exhibited remarkable genomic similarity, differing by a maximum of nine SNPs, and belonging to the same *S.* Enteritidis sequence type, ST11. While lacking traditional antimicrobial resistance genes, they shared a conserved repertoire of efflux pumps and regulatory proteins associated with resistance mechanisms [43] (S2 Fig), coupled with analogous profiles of virulence genes (S4 Fig) and the plasmid replicons *IncFII(S)-1* and *IncFIB(S)-1* (S3 Fig).

The genetic concordance among isolates from the case (a sick child) and its immediate environment highlights the potential role of the environment as a reservoir for NTS, acting as a focal point for sustained exposure and reinfection within the community, especially for those living in resource-limited areas, where poor housing conditions and inadequate sanitation infrastructure increase the risks of NTS transmission [2,3,6]. Our findings correspond with the comprehensive review by Oludairo et al. [44], which identified water and soil as critical pathways for human exposure, emphasizing the complex role of environmental reservoirs in infection cycles. This perspective enhances the understanding of infection dynamics in endemic regions and supports the hypothesis that environmental reservoirs may significantly contribute to sustaining infection cycles, particularly in domestic settings within peri-urban informal settlements.

Our study revealed consistently high detection rates of NTS throughout the year in Mukuru informal settlements, with noticeable peaks during the wet months of April to June and October to December. Despite these seasonal fluctuations, statistical analysis showed no significant variation (Z-scores < 1.96) in detection rates, suggesting that the burden of NTS remains relatively stable year-round, irrespective of rainfall patterns. The absence of marked seasonal trends in the detection of NTS further emphasizes the status of Mukuru informal settlement as a high-risk area, particularly for children under the age of five [2,3,39]. Overcrowding, inadequate sanitation infrastructure, improper sewage disposal, and limited access to safe drinking water [2,3,6] are persistent challenges in informal settlements that create an environment conducive to the continuous transmission of NTS, thereby sustaining its prevalence throughout the year.

Whilst this study offers important insights into the role of environmental contamination in the transmission of NTS, several limitations need to be acknowledged to ensure a balanced interpretation of the findings. A key limitation is the detection of NTS DNA without isolating viable bacterial strains. This approach, while useful in detecting the presence of NTS, does not allow us to definitively quantify the environmental burden of the bacteria. As such, the results should be viewed as indicative of NTS persistence in the environment rather than an exact measure of NTS load or prevalence. Additionally, this study did not perform source tracking of NTS pathogens, which prevents us from conclusively determining the direction of association. While it is reasonable to hypothesize that environmental contamination may serve as a reservoir for infection, we cannot rule out the possibility that infected children could be introducing NTS into their surroundings, thereby contributing to environmental contamination. This bidirectional relationship has been observed in other studies on enteric pathogens, where both environmental exposure and human shedding play significant roles in disease transmission[39,45]. Furthermore, our study did not account for other critical environmental and anthropogenic factors that could influence NTS survival and proliferation, such as temperature, humidity, and human activities [8,46,47]. Despite these limitations, the study underscores a crucial but often-overlooked contribution of environmental contamination to the persistence and transmission of NTS, particularly in informal settlements where poor living conditions and inadequate sanitation practices exacerbate the risk of infection.

## Conclusion

This study demonstrates that environmental contamination plays a crucial role in sustaining NTS infections in informal settlements. Case households exhibited significantly higher contamination rates, with open drains, drinking water, and soil emerging as potential NTS transmission pathways. The persistence of NTS throughout the year, coupled with the isolation of genetically similar *S. enteritidis* ST11 in both clinical and environmental samples, highlights the plausible role of household environments as reservoirs for NTS, perpetuating continuous exposure and reinfection cycles in these vulnerable communities.

The findings from this study underscore the critical need for continuous surveillance of NTS in environmental and community settings to better understand its ecology and transmission, particularly in informal settlements where infrastructural challenges heighten disease risks. Addressing this burden requires a multi-sectoral approach that integrates improved sanitation infrastructure, systematic environmental monitoring, and evidence-based community interventions. By combining microbiological surveillance, genomic epidemiology, and policy-driven sanitation improvements, targeted public health efforts can break the chain of transmission and mitigate the persistent threat of NTS in resource-limited settings.

## Supporting information

S1 Table Comparative Analysis of Single Nucleotide Polymorphisms (SNPs) in *Salmonella* Enteritidis Isolates from Index Cases and Environmental Samples. This table presents genomic characteristics of *Salmonella* Enteritidis isolates recovered from an index case (a sick child) during acute illness and follow-up periods, alongside isolates from environmental samples. The table includes isolate sequence IDs, serotype, sequence type, and the number of single nucleotide polymorphisms (SNPs) relative to the reference genome (Salmonella Typhimurium LT2, ASM694v2). Additionally, the table provides information on plasmid replicons (*IncFII(S)_1* and *IncFIB(S)_1),* the presence of the *aac(6’)-Iy* gene, and the total number of identified virulence factors in each isolate. Environmental samples include isolates from drinking water and effluent, highlighting potential reservoirs for transmission.
(XLS)

S2 Table Functional Classification of Detected Virulence Genes from Environmental and Clinical Samples, Including Genes Encoded by Salmonella Pathogenicity Islands (SPI-1 and SPI-2). The virulence genes were categorized by their functional roles in bacterial pathogenicity. These include processes such as adhesion, invasion, toxin production, immune evasion, iron acquisition, and stress responses, as well as genes linked to plasmid-associated virulence and outer membrane proteins. Genes encoded by Salmonella Pathogenicity Islands (SPI-1 and SPI-2) highlight their importance in invasion and intracellular survival. This classification provides insight into the diverse mechanisms employed by Salmonella to infect hosts and persist across different ecological niches.
(DOCX)

S2 Fig Antimicrobial Resistance Associated Genes. Heatmap illustrating the presents and the distribution of antimicrobial resistance-associated genes in *Salmonella* Enteritidis isolates from the index case (a sick child) and those from its immediate environment. Each row represents a detected gene, while columns correspond to specific sample types. The color gradient, ranging from yellow to red, signifies gene percent coverage, with red indicating higher coverage. *Samples are classified into environmental and clinical categories, highlighting potential divergence in antimicrobial resistance of Salmonella* Enteritidis isolates from the index case (sick child) and those from their immediate environment. Isolates labeled 030353_D_cult and 030353_E_Cult correspond to samples recovered from the index case’s drinking water and their homestead effluent/ open drains, respectively. The number 030353_Case refers to the first isolate obtained from the index case during the acute disease period, while 030353_F1 and 030353_F3 represent isolates recovered from the index case during the first and third follow-ups, respectively.
(TIF)

S3 Fig Plasmid Replicons Genes. Heatmap illustrating the presents and the distribution of plasmid replicons in *Salmonella* Enteritidis isolates from the index case (a sick child) and those from its immediate environment. Each row represents a detected gene, while columns correspond to specific sample types. The color gradient, ranging from yellow to red, signifies gene percent coverage, with red indicating higher coverage. *Samples are classified into environmental and clinical categories,* the diversity of plasmid replicons in *Salmonella* Enteritidis isolates from the index case (sick child) and those from their immediate environment. Isolates labeled 030353_D_cult and 030353_E_Cult correspond to samples recovered from the index case’s drinking water and their homestead effluent/ open drains, respectively. The number 030353_Case refers to the first isolate obtained from the index case during the acute disease period, while 030353_F1 and 030353_F3 represent isolates recovered from the index case during the first and third follow-ups, respectively.
(TIF)

S4 Fig Virulence-Associated Genes. Heatmap illustrating the presents and the distribution of virulence-associated genes in *Salmonella* Enteritidis isolates from the index case (a sick child) and those from its immediate environment. Each row represents a detected gene, while columns correspond to specific sample types. The color gradient, ranging from yellow to red, signifies gene percent coverage, with red indicating higher coverage. *Samples are classified into environmental and clinical categories, highlighting potential divergence and similarities in virulence-associated genes of Salmonella* Enteritidis isolates from the index case (sick child) and those from their immediate environment. Isolates labeled 030353_D_cult and 030353_E_Cult correspond to samples recovered from the index case’s drinking water and their homestead effluent/ open drains, respectively. The number 030353_Case refers to the first isolate obtained from the index case during the acute disease period, while 030353_F1 and 030353_F3 represent isolates recovered from the index case during the first and third follow-ups, respectively.
(TIF)
