# Supplementary material for: Environmental persistence of nontyphoidal Salmonella in an urban informal settlement in Nairobi, Kenya
Source: PLoS One. 2025 Apr 28;20(4):e0321760. doi: 10.1371/journal.pone.0321760 (PMC12036858; doi:10.1371/journal.pone.0321760)
Supplement: S2 Table — The virulence genes were categorized by their functional roles in bacterial pathogenicity. These include processes such as adhesion, invasion, toxin production, immune evasion, iron acquisition, and stress responses, as well as genes linked to plasmid-associated virulence and outer membrane proteins. Genes encoded by Salmonella Pathogenicity Islands (SPI-1 and SPI-2) highlight their importance in invasion and intracellular survival. This classification provides insight into the diverse mechanisms employed by Salmonella to infect hosts and persist across different ecological niches. (DOCX) [file pone.0321760.s002.docx]

| **Function** | | **Detected Virulence Genes** |
| --- | --- | --- |
| **Adhesion related genes** | | *csgA-G, fimC-I, lpfA-E, misL, pefA-D, sinH* |
| **Invasion related genes** | | *invA-J, sipA-D, sptP, orgA, prgH-K* |
| **Toxin Production related genes** | | *sopA-D, sopD2, sopE2, slrP* |
| **Immune Evasion related genes** | | *avrA, sodCI, mig-14* |
| **Iron Uptake and Metabolism related genes** | | *entA-B, fepG* |
| **Type III Secretion System (T3SS) associated genes** | **SPI-1** | *spaO-S, orgB-C, sicA, sicP* |
|  | **SPI-2** | *spiC/ssaB, ssaC-V, sseA-L, pipB-B2, sifA-B, sscA-B, sspH2, steA-C, sseK1* |
| **Salmonella Plasmid-associated Virulence related genes** | | *spvB, spvC, spvR* |
| **Stress Response related genes** | | *mgtB-C, ratB* |
| **Outer Membrane Proteins related genes** | | *ompA, rck* |
