# Supplementary figures and images for: Environmental persistence of nontyphoidal Salmonella in an urban informal settlement in Nairobi, Kenya
Source: PLoS One. 2025 Apr 28;20(4):e0321760. doi: 10.1371/journal.pone.0321760 (PMC12036858; doi:10.1371/journal.pone.0321760)

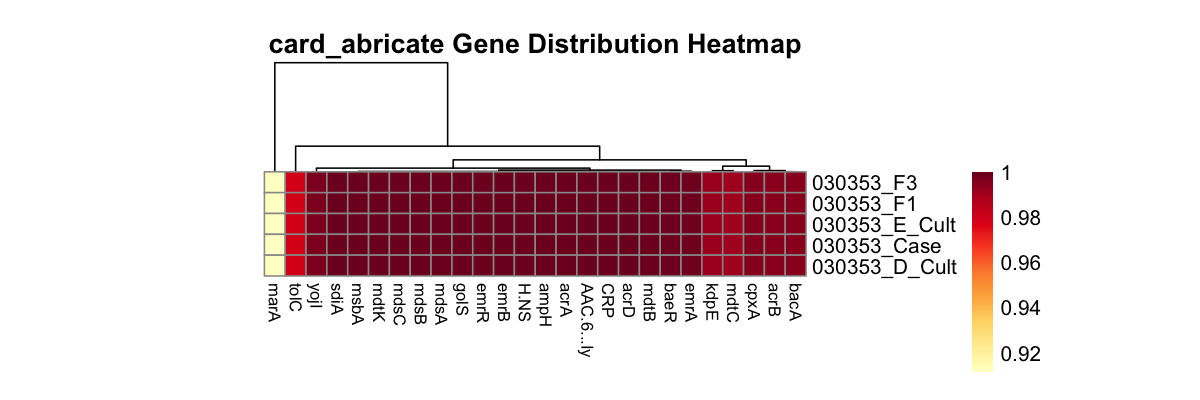

Supplement: S2 Fig — Heatmap illustrating the presents and the distribution of antimicrobial resistance-associated genes in Salmonella Enteritidis isolates from the index case (a sick child) and those from its immediate environment. Each row represents a detected gene, while columns correspond to specific sample types. The color gradient, ranging from yellow to red, signifies gene percent coverage, with red indicating higher coverage. Samples are classified into environmental and clinical categories, highlighting potential divergence in antimicrobial resistance of Salmonella Enteritidis isolates from the index case (sick child) and those from their immediate environment. Isolates labeled 030353_D_cult and 030353_E_Cult correspond to samples recovered from the index case’s drinking water and their homestead effluent/ open drains, respectively. The number 030353_Case refers to the first isolate obtained from the index case during the acute disease period, while 030353_F1 and 030353_F3 represent isolates recovered from the index case during the first and third follow-ups, respectively. (TIF) [file pone.0321760.s003.tif]

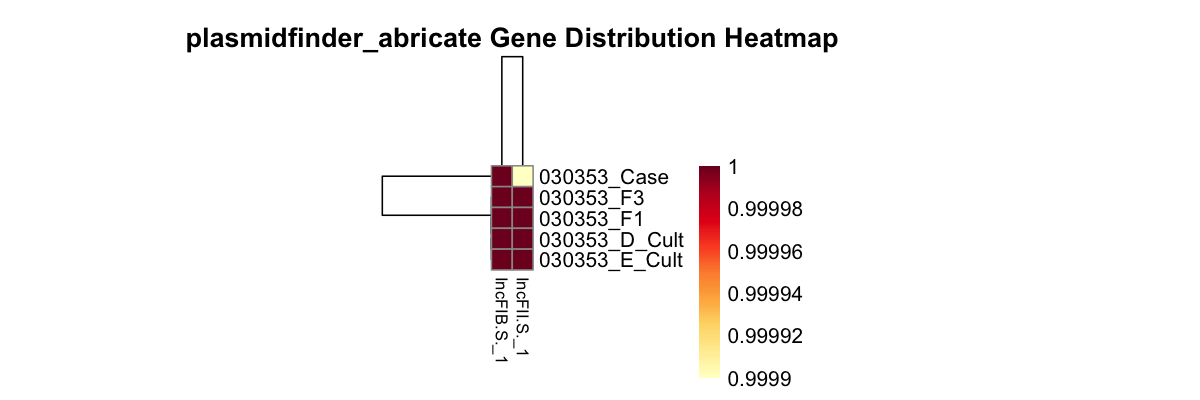

Supplement: S3 Fig — Heatmap illustrating the presents and the distribution of plasmid replicons in Salmonella Enteritidis isolates from the index case (a sick child) and those from its immediate environment. Each row represents a detected gene, while columns correspond to specific sample types. The color gradient, ranging from yellow to red, signifies gene percent coverage, with red indicating higher coverage. Samples are classified into environmental and clinical categories, the diversity of plasmid replicons in Salmonella Enteritidis isolates from the index case (sick child) and those from their immediate environment. Isolates labeled 030353_D_cult and 030353_E_Cult correspond to samples recovered from the index case’s drinking water and their homestead effluent/ open drains, respectively. The number 030353_Case refers to the first isolate obtained from the index case during the acute disease period, while 030353_F1 and 030353_F3 represent isolates recovered from the index case during the first and third follow-ups, respectively. (TIF) [file pone.0321760.s004.tif]

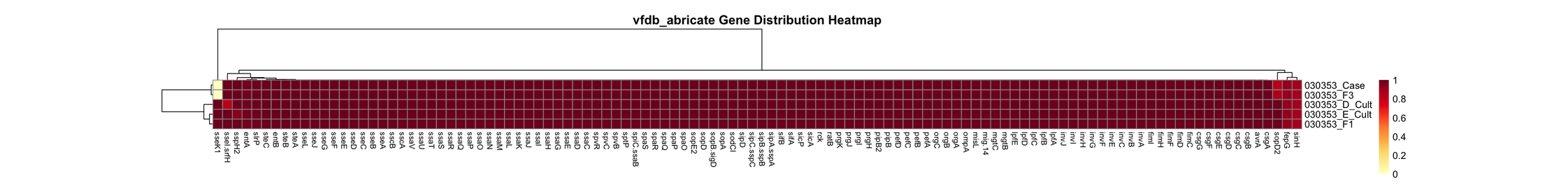

Supplement: S4 Fig — Heatmap illustrating the presents and the distribution of virulence-associated genes in Salmonella Enteritidis isolates from the index case (a sick child) and those from its immediate environment. Each row represents a detected gene, while columns correspond to specific sample types. The color gradient, ranging from yellow to red, signifies gene percent coverage, with red indicating higher coverage. Samples are classified into environmental and clinical categories, highlighting potential divergence and similarities in virulence-associated genes of Salmonella Enteritidis isolates from the index case (sick child) and those from their immediate environment. Isolates labeled 030353_D_cult and 030353_E_Cult correspond to samples recovered from the index case’s drinking water and their homestead effluent/ open drains, respectively. The number 030353_Case refers to the first isolate obtained from the index case during the acute disease period, while 030353_F1 and 030353_F3 represent isolates recovered from the index case during the first and third follow-ups, respectively. (TIF) [file pone.0321760.s005.tif]
